# Aboveground Whitefly Infestation Modulates Transcriptional Levels of Anthocyanin Biosynthesis and Jasmonic Acid Signaling-Related Genes and Augments the Cope with Drought Stress of Maize

**DOI:** 10.1371/journal.pone.0143879

**Published:** 2015-12-02

**Authors:** Yong-Soon Park, Dong-Won Bae, Choong-Min Ryu

**Affiliations:** 1 Molecular Phytobacteriology Laboratory, KRIBB, Daejeon, 305–806, South Korea; 2 Agricultural Microbiology Division, National Academy of Agricultural Science, RDA, Wanju, 565–851, South Korea; 3 Central Instrument Facility, Gyeongsang National University, Jinju, 660–701, South Korea; 4 Biosystems and Bioengineering Program, University of Science and Technology (UST), Daejeon, 305–350, South Korea; Gyeongnam National University of Science and Technology, REPUBLIC OF KOREA

## Abstract

Up to now, the potential underlying molecular mechanisms by which maize (*Zea mays* L.) plants elicit defense responses by infestation with a phloem feeding insect whitefly [*Bemisia tabaci* (Genn.)] have been barely elucidated against (a)biotic stresses. To fill this gap of current knowledge maize plants were infested with whitefly and these plants were subsequently assessed the levels of water loss. To understand the mode of action, plant hormone contents and the stress-related mRNA expression were evaluated. Whitefly-infested maize plants did not display any significant phenotypic differences in above-ground tissues (infested site) compared with controls. By contrast, root (systemic tissue) biomass was increased by 2-fold by whitefly infestation. The levels of endogenous indole-3-acetic acid (IAA), jasmonic acid (JA), and hydrogen peroxide (H_2_O_2_) were significantly higher in whitefly-infested plants. The biosynthetic or signaling-related genes for JA and anthocyanins were highly up-regulated. Additionally, we found that healthier plants were obtained in whitefly-infested plants under drought conditions. The weight of whitefly-infested plants was approximately 20% higher than that of control plants at 14 d of drought treatment. The drought tolerance-related genes, *ZmbZIP72*, *ZmSNAC1*, and *ZmABA1*, were highly expressed in the whitefly-infected plants. Collectively, our results suggest that IAA/JA-derived maize physiological changes and correlation of H_2_O_2_ production and water loss are modulated by above-ground whitefly infestation in maize plants.

## Introduction

Plants protect themselves *via* their defensive mechanisms against diverse biotic and abiotic stresses [1]. Approximately half a million insect species feed on plants, which constitutes one of the greatest biotic stresses [2]. Plant defense strategies against insect herbivory include enforcement of physical barriers and direct and indirect elicitation of inducible defenses [1]. Insects have evolved their feeding styles and behaviors during the last 350 million years to overcome plant defense strategies as well [3]. Herbivorous insects are classified into two major groups according to the mouth structure and the feeding type. The chewing insects have typically chewing mouthparts (i.e. grasshoppers and beetles), and tear off and digest the disrupted plant tissues [4]. On the other hand, the piercing and sucking insects use a stylet to pierce plant tissues and suck out the liquids from plant tissues [5]. No visible damage to plant tissues is observed as a result of stylet piercing, and plant behaviors in response to piercing insects are not similar to those caused by mechanical wounding of plant tissues [5]. Aphids and whiteflies belong to the piercing and sucking insect herbivores group.

Whitefly [*Bemisia tabaci* (Genn.)] is a small phloem-sucking insect. Whiteflies are classified into two independent subfamilies by means of origin, and approximately 1,500 species are widely distributed in warm, tropical, and subtropical areas [6,7]. There is compelling evidence that whitefly infestation induces plant defense responses such as the accumulation of defense compounds, emission of volatile signals [8], activation of salicylic acid (SA) and jasmonic acid (JA)/ethylene dependent pathways [9,10], and up-regulation of several pathogenesis-related (PR) proteins [7, 11,12].

Maize (*Zea mays* L.) is one of the most important cereal crops [13]. Due to the agricultural and economic importance of maize, many researchers are developing novel strategies to enhance crop yield and resistance to multiple biotic and abiotic stresses. Recent efforts are elucidating the precise role of pathogens and chewing insects in eliciting disease resistance in maize [14,15]. Whitefly infestation has been a global issue since the late 1980s [16,17]. Although there have been reports demonstrating interactions between *Bemisia tabaci* and maize plants [18,19], a comprehensive study of how whitefly piercing affects maize defense responses and development is needed.

In this study, to broaden the knowledge on maize-whitefly interactions we investigated the effect of whitefly infestation on maize physiological development and drought stress using molecular and biochemical approaches. Our findings can somehow impact on the precise elucidation of maize molecular mechanism behinds by whitefly infestation, resulting in increasing the cope with drought of maize plants.

## Materials and Methods

### Maize plant growth and whitefly treatments

Maize seeds (*Zea mays* L. cv. Mibaek^2nd^) were surface-sterilized with 6% sodium hypochlorite, washed three times with sterile distilled water (SDW), and sown in soilless potting medium (Punong, Co. Ltd., Gyeongju, South Korea) at 28 ± 2°C under natural greenhouse facility conditions (KRIBB, Daejeon, South Korea). The V2 stage maize seedlings were transferred to plastic containers (65 × 70 × 80 cm). Transferred plants were constantly infested with naturally occurred whiteflies [*Bemisia tabaci* (Genn.)], and were cultivated for 4 weeks in the plastic containers under greenhouse conditions. Approximately 15 whiteflies were constantly feeding on the leaves per plant. Ten biological replicates were used for each experiment, and each experiment was repeated three times with similar results.

For gene expression analysis, maize seeds were sown and grown until the V2 stage under the same environmental conditions described previously. Maize seedlings were infested with whitefly and harvested at 0, 6, 12, 24, 48, 72, and 96 h after the start of infestation. Control plants were not infested with whitefly and harvested at the same time points along with whitefly-infested plants. To avoid cross-contamination of control plants with whitefly-infested plants, we incubated and infested treated plants inside separate masked nets. Leaves and roots were separately harvested at designated time points and immediately frozen with liquid N_2_ for extraction of total RNA for gene expression profiling. Approximately 15 whiteflies were feeding on the leaves per plant.

### Plant development performance

To assess whether whitefly infestation affected plant fitness, height, leaf fresh weight, stem thickness, stem width, and developmental stage were measured after 4 weeks of whitefly infestation under the same conditions described previously. In addition, root architecture (primary root length, numbers of seminal roots and crown roots, and total root weight) and root anthocyanin pigmentation were monitored in whitefly-infested and control plants.

### Total RNA extraction and cDNA synthesis

The V2 stage maize seedlings were infested with whitefly, and leaf and root samples were separately harvested at 0, 6, 24, 48, 72, and 96 h. Frozen samples were ground with a sterilized mortar and pestle using liquid N_2_. Total RNA was isolated from ground tissues using a previously published method [20]. Briefly, 100 mg of ground tissues were added to 1 ml of TRI reagent, and subsequent extraction methods were according to the manufacturer’s instructions (NanoHelix Co., Ltd., Daejeon, South Korea). RNA quality and quantity were confirmed by gel electrophoresis and a Nanodrop spectrophotometer (ND-1000, DE, USA) before use in subsequent analyses. Synthesis of first-strand cDNA was performed from 1 μg of total RNA as a template with oligo-dT primer (Qiagen, CA, USA), dNTPs (Qiagen, CA, USA), and Moloney murine leukemia virus reverse transcriptase (MMLV-RT; Enzynomics, Daejeon, South Korea).

### Quantitative (q)-RT-PCR

To ensure that equal amounts of RNA were analyzed for each sample, semi-quantitative RT-PCR was performed with a maize housekeeping gene (*ZmGapc*) under the following reaction conditions: 95°C for 5 min; 27 cycles of 95°C for 30 sec, 55°C for 30 sec, and 72°C for 1 min; and 72°C for 5 min. PCR products were separately loaded on 1.2% agarose gels for electrophoresis.

To test the expression of candidate genes by qRT-PCR, each reaction mixture contained 5 μl of 2× Brilliant SYBR Green QPCR Master Mix (Bio-Rad, CA, USA), cDNA, 0.5 μM of each primer, and 10 μl final reaction volume. Reactions were amplified in a Chromo4 Real-Time PCR System (Bio-Rad, CA, USA) under the following conditions for each gene: 95°C for 10 min, and 44 cycles of 95°C for 30 sec, 60°C for 30 sec, and 72°C for 30 sec. The relative expression of each gene was normalized to that of *ZmGapc*. All primers used in this study are listed in S1 **Table**.

### Quantification of levels of endogenous plant hormones

Five replicate samples per treatment were analyzed to quantify free-SA, total JA, IAA, and ABA levels in leaf and root at 48 h after whitefly infestation. The preparation of samples and procedure of analysis was followed previously [21]. Briefly, samples were grounded into a fine powder in liquid nitrogen. Approximately 50 mg of finely grounded samples was dissolved in 500 μl of extraction solvent [2-propanol/H_2_O/HCl (2:1:0.002, v/v/v)] and were shaken at 100 rpm at 4°C for 30min. The each sample was added 1 ml dichloromethane, shaken one more time, and centrifuged at 13,000g for 5 min. About 900 μl from lower phase was transferred, dried, and resolved in 100 μl methanol. Internal standard solution was prepared at a concentration of 50 ng/ml in methanol. Samples (10 μl) were injected onto Esclipe at plus C18 (4.6 X 50 mm, 3.5 μm) column (Agilent, Santa Clara, CA, USA), and analyzed on Qtrap 3200 mass spectrometer (AB Sciex, Concord, Canada) coupled to Agilent 1100 HPLC system (Agilent, Santa Clara, CA, USA) at a flow rate 500 μl/min.

### Drought-stress assay

The V2 stage maize seedlings were exposed to whitefly or control for 4 weeks in plastic containers as followed above, and then plants were not applied with water for cultivating for treatment of drought stress. Plant fresh weights were measured before stopping water. After stopping water for whitefly-infested and control plants, plant fresh weights were measured at designated time points. The relative plant fresh weight at each time point was calculated and normalized with respect to the original weight (0 d). This normalized relative weight was quantified and displayed at each time point. The five biological replicates per treatment at each time point were monitored for assessment of drought stress.

### Detection of H_2_O_2_ by DAB staining

The procedure for whitefly infestation and application of drought was followed as described above. We performed DAB staining to detect hydrogen peroxide (H_2_O_2_) In brief, the maize leaves were excised 2 d after whitefly infestation and 8 d after drought stress from whitefly-infested plants, and were placed in DAB solution (3,3-diaminobenzidine-HCl, pH 3.8, 1 mg/ml) overnight at room temperature. The segments of leaves were placed in boiling ethanol until completely de-stained. The five replicates per treatment at each time point were examined.

### Statistical analysis

Analysis of variance for experimental datasets was performed using JMP software v5.0 (SAS Institute, Cary, NC, USA) for all data. Significant treatment effects were determined by the magnitude of the *F* value (*P* = 0.05). When a significant *F* test was obtained, separation of means was accomplished by Fisher’s protected least significant difference (LSD) at *P* = 0.05.

## Results

### Above-ground whitefly infestation modulates plant growth and development

**Fig 1:**
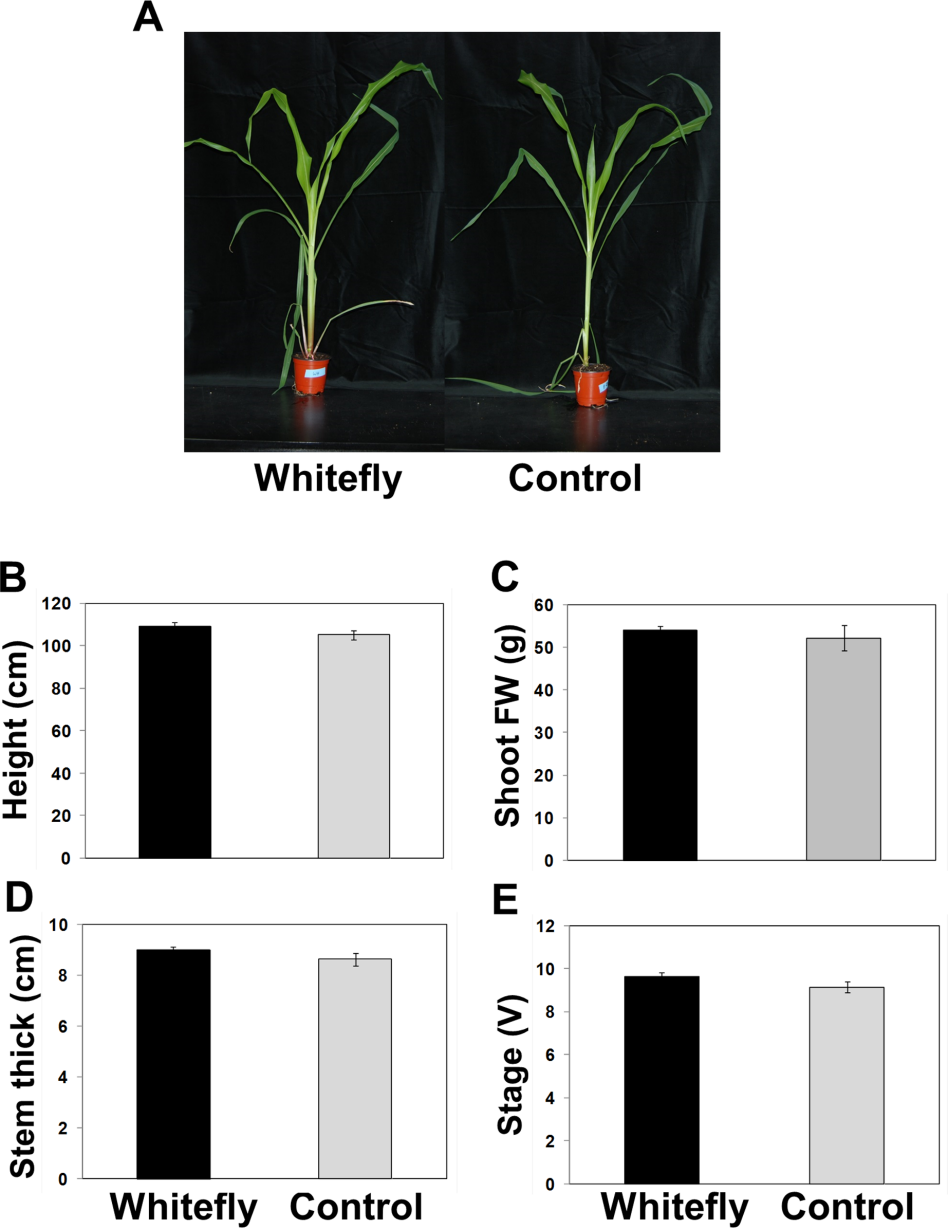
Shoot phenotypes of whitefly-infested plants. The V2 stage maize seedlings were infested with whitefly for 4 weeks. Effect of whitefly infestation on whole plant phenotypic physiology. **A**. The photograph was taken at 4 weeks after whitefly infestation (left) or control treatment (right). Plant height (**B**), shoot fresh weight (**C**), stem thickness (**D**), and developmental stage (**E**) were measured 4 weeks after whitefly infestation or control treatment. Ten biological replicates per treatment were used for the experiment. Bars represent the mean value of standard error. Experiments were repeated three times with similar results.

To investigate this phenomenon in monocot plant species, we selected maize inbred line (*Zea mays* L. cv. Mibaek^2nd^) that was cultivated in South Korea. In order to investigate how whitefly infestation affected maize growth and developments, we monitored above-ground plant phenotypes after whitefly infestation or control treatment. No significant difference was visually identified between whitefly-infested and control plants after 4 weeks after treatment (**Fig 1A**). The mean height of individual plants (from soil level to highest leaf tip) was 109 and 105 cm in size in whitefly-infested and control plants, respectively (**Fig 1B**). Approximately 53 g of shoot fresh weight per plant was measured for whitefly-infested and control plants (**Fig 1C**). Stem thickness (**Fig 1D**) and stem width (**S1 Fig**) were statistically equivalent in whitefly-infested plants and control plants. As expected, these results indicate that there is no significant difference in the foliar development and growth of whitefly-infested and control maize plants (**Fig 1E**).

**Fig 2:**
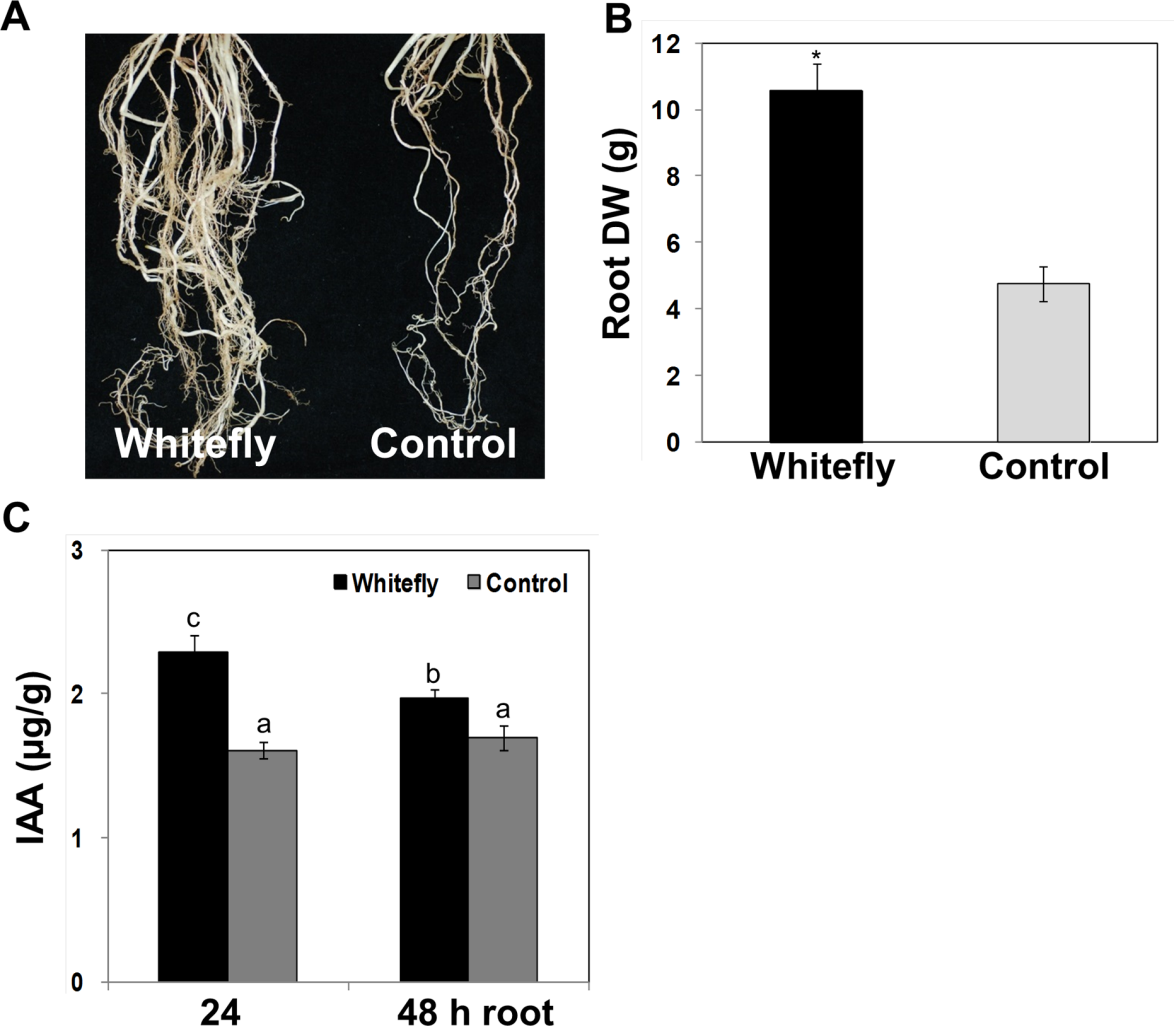
Stimulation of total root biomass production by whitefly infestation. The V2 stage maize seedlings were infested with whitefly for 4 weeks. **A**. The representative picture was taken 4 weeks after whitefly infestation (left) or control treatment (right). **B**. Root dry weight was measured at 4 weeks after infestation or control treatment. Ten biological replicates per treatment were used for the experiment. **C**. Endogenous IAA was measured in maize roots at 24 and 48 h after whitefly infestation. Bars represent the mean value of standard error, and an asterisk and different letters above the graph indicate a significant difference between treatments (*P* = 0.05). Experiments were repeated three times with similar results.

Despite of no significant alteration in above-ground (foliar) growth and developments in between treatments, we then carefully monitored the effect of whitefly infestation on growth and development of below-ground maize tissues relative to that of control plants. Notably, whitefly infestation enhanced the growth of seminal and lateral roots (**Fig 2A**). In agreement with increasing of lateral and seminar root numbers, the total root dry weight was two times higher in whitefly-infested plants than in control plants (**Fig 2B**). The endogenous IAA levels were statistically higher in whitefly-infested plants when compared to control (**Fig 2C**).

**Fig 3:**
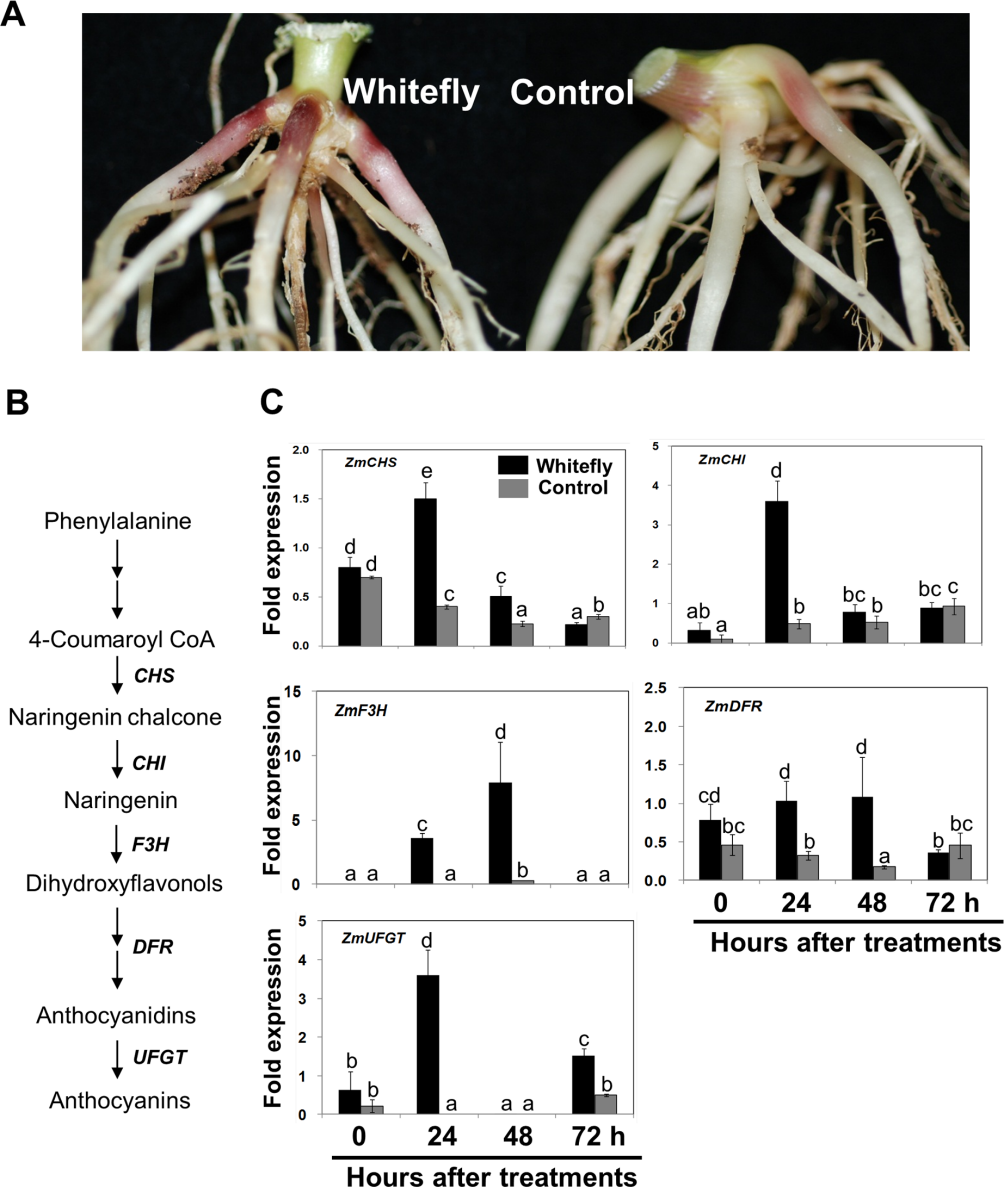
Whitefly infestation induces anthocyanin pigmentation in roots. **A**. Maize seedlings were exposed to whitefly for 4 weeks as described in Fig 1. Higher anthocyanin levels accumulated in roots of whitefly-infested plants than in control plants. Ten biological replicates were used in this experiment. Experiments were repeated three times with similar results. **B**. Simplified schematic representation of anthocyanin biosynthetic pathway from phenylalanine, modified from previous reports [29,49]. Enzyme names are as follows: *CHS*, *chalcone synthase*; *CHI*, *chalcone isomerase*; *F3H*, *flavonone-3-hydroxylase*; *DFR*, *dihydroflavonol reductase*; *UFGT*, *UDP-flavonoid glucosyl transferase*. **C**. Maize gene expression levels were quantified by qRT-PCR. Transcript levels of each gene were relatively quantified and normalized with *ZmGapc*. Bars represent the mean value of standard error, and different letters above the graph indicate significant differences between treatments and time points (*P* = 0.05).

Interestingly, significant accumulation of red pigment on crown part of the whitefly infested plant was observed (**Fig 3A**). The chemical property was identified the pigment as anthocyanins. In spite of tremendous evidence for the role of anthocyanin in most plant species, the underlying mechanism by which whitefly infestation facilitated needs to have further investigation. We tested if whitefly infestation activated anthocyanin biosynthesis in maize roots by quantifying the transcript levels of several anthocyanin biosynthetic genes in whitefly-infested and control plants. The concise anthocyanin biosynthetic pathway was presented in **Fig 3B**. The expression patterns of the early biosynthetic genes *chalcone synthase* (*ZmCHS*), *chalcone isomerase* (*ZmCHI*), and *flavanone 3-hydroxylase* (*ZmF3H*) showed that mRNA levels were up-regulated at 24 h after infestation compared with those of control plants and the initial time point (at 0 h), among which *ZmF3H* was significantly up-regulated at both 24 and 48 h (**Fig 3C**). The expression pattern of the late biosynthetic genes *dihydroflavonol reductase* (*ZmDFR*) and *UDP-flavonoid glucosyl transferase* (*ZmUFGT*) showed that although *ZmDFR* mRNA level was relatively higher at 24 and 48 h after whitefly infestation, these levels were not significantly up-regulated in whitefly-infested roots when compared to the initial time point (at 0 h). However, *ZmUFGT* was strongly up-regulated in maize roots at 24 and 72 h after infestation (**Fig 3C**). Based on these results, we conclude that four out of five genes may play pivotal roles in endogenous anthocyanin accumulation after whitefly feeding.

### Upregulation of JA biosynthesis gene and accumulation of JA in whitefly-infested leaf and root

**Fig 4:**
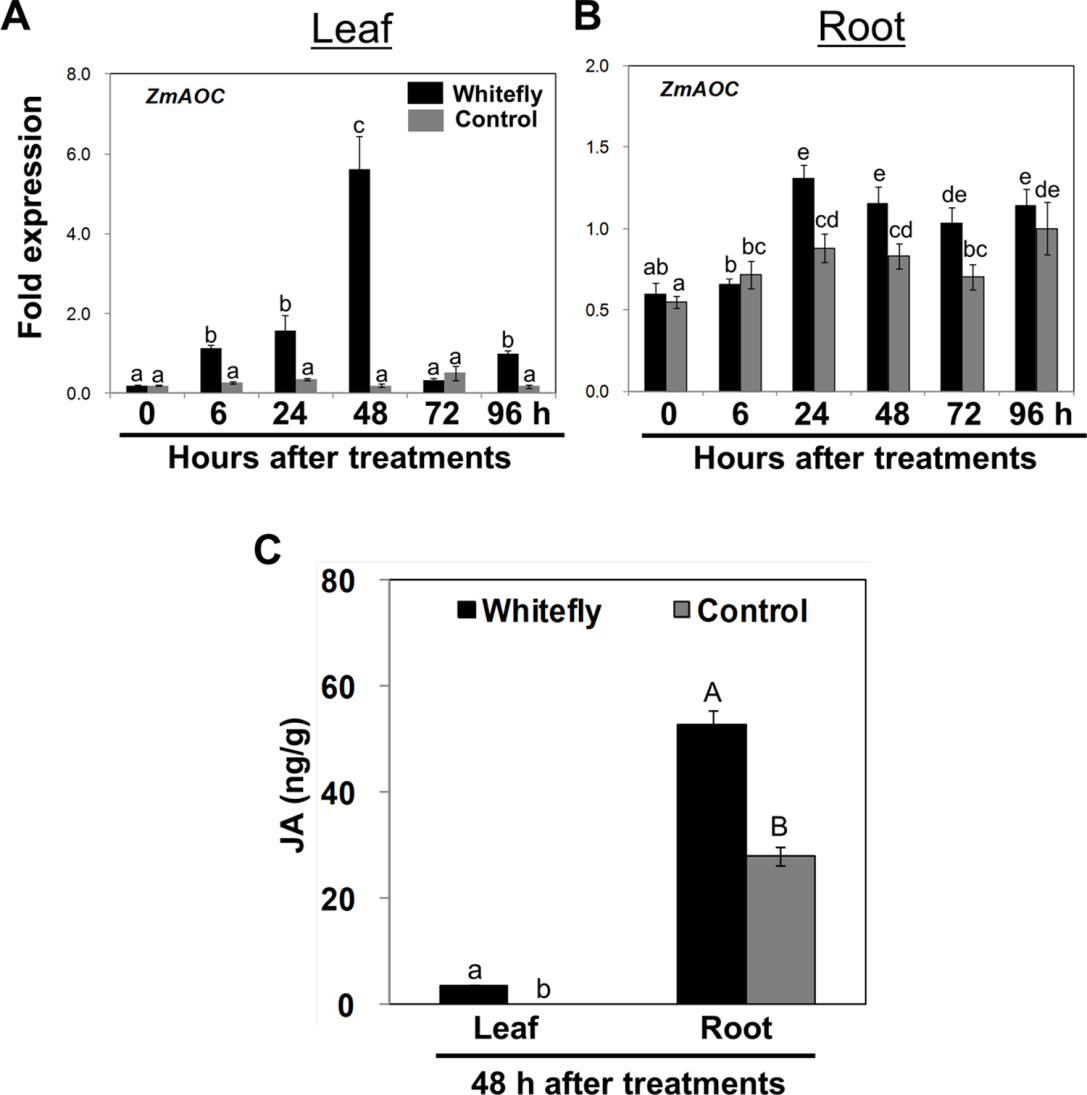
Transcript profile of *ZmAOC* and quantification of endogenous JA in leaf and root after whitefly infestation. The V2 stage maize seedlings were infested with whitefly and harvested at 0, 6, 24, 48, 72, and 96 h after infestation or control treatment. The expression levels of maize gene *ZmAOC* was quantified by qRT-PCR in leaf (**A**) and root (**B**). The transcript level of each gene was relatively quantified and normalized using *ZmGapc*. **C**. Accumulation of endogenous total-JA was measured at 48 h after whitefly infestation. Bars represent the mean value of the standard error, and different letters above the graph indicate significant differences between treatments and time points (*P* = 0.05).

To obtain molecular evidence, we performed qRT-PCR of maize genes implicated in biosynthesis or signaling in phytohormone-dependent pathways in leaf and root of whitefly-infested and control-treated maize plants (data not shown). In response to whitefly feeding, out of other stress hormone-related genes, the JA biosynthesis gene *allene oxide cyclase* (*ZmAOC*) [22] was significantly up-regulated in leaf by up to 28-fold at 48 h after infestation (**Fig 4A**), and was up-regulated in root from 24–72 h after infestation (**Fig 4B**).

The significant up-regulation of JA biosynthetic gene (*ZmAOC*) led us to measure total JA level in leaf and root of whitefly-infested and control plants. The V2 stage maize seedlings were exposed to whitefly for 48 h before harvesting and measuring JA. The results showed that JA was only detected in whitefly-infested leaf but not in control leaf (**Fig 4C**), and approximately two times higher JA levels were detected in whitefly-infested roots (**Fig 4C**). However, no overexpression of SA biosynthesis gene and accumulation of SA were observed in whitefly-infested leaf and root (**S2 Fig**).

### Whitefly infestation induces drought-stress tolerance in maize

**Fig 5:**
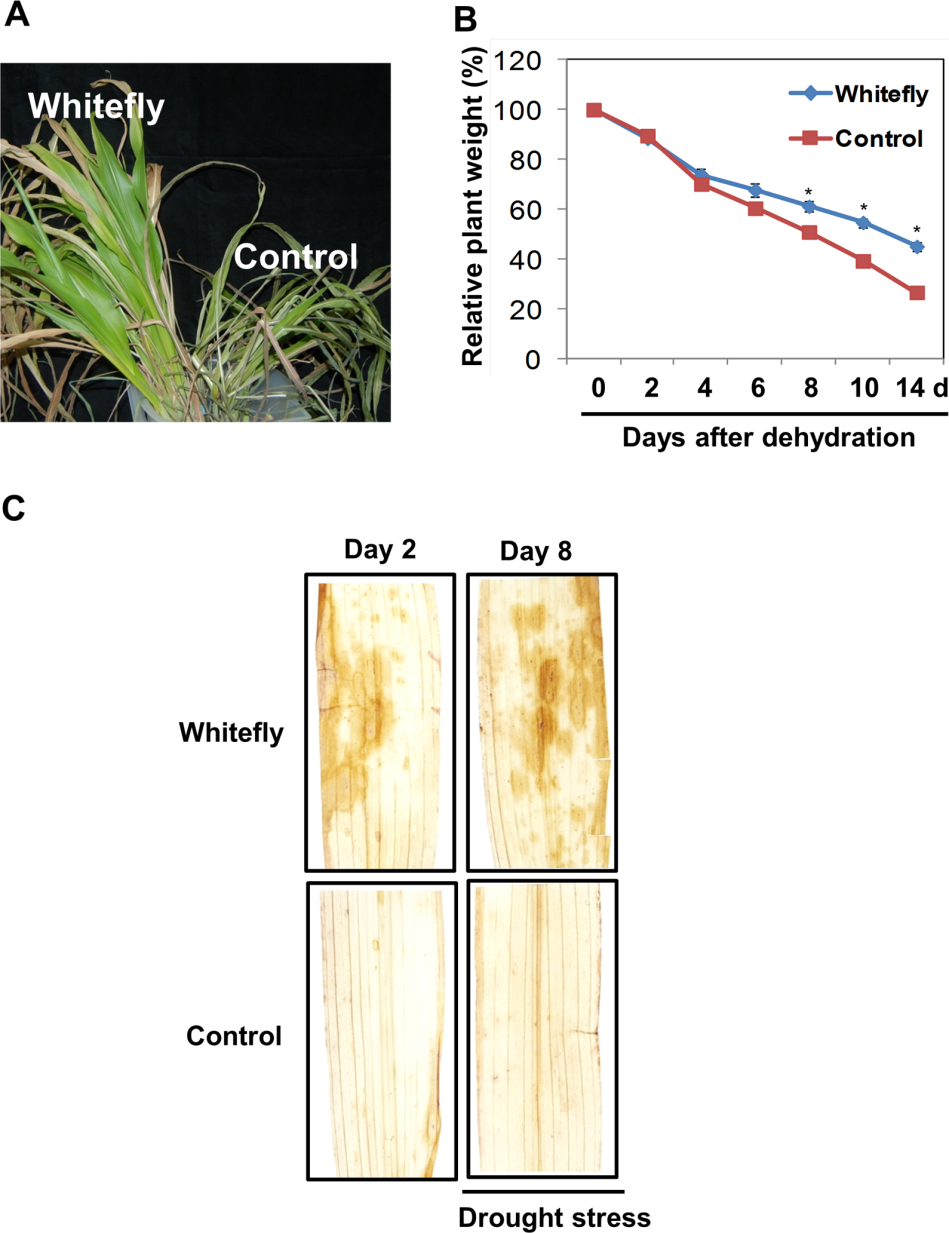
Whitefly infestation enhances drought tolerance in maize. **A**. The V2 stage maize seedlings were infested with whitefly for 4 weeks, and then infested plants were not watered for 2 weeks. Infested plants (left side) were relatively healthier than control plants (right side). **B**. Relative plant weight was measured at designated time points after withholding water and compared to the initial weight at 0 d (the day when water was first withheld). Blue and red lines indicate mean relative weight at each time point in whitefly-infested and control plants, respectively. Asterisks above the blue line indicate significant differences between treatments at 8–14 d (*P* = 0.05). **C**. Hydrogen peroxide was detected in whitefly-infested plants before and after drought stress. Five biological replicates per treatment at each time point were measured. Experiments were repeated twice with similar results.

To investigate whether whitefly infestation may affect on drought stress the V2 stage maize seedlings were treated with whitefly infestation or control treatment for 4 weeks, and subsequently water was withheld. Interestingly, the whitefly-infested plants were relatively healthier than control plants after drought treatment (**Fig 5A**). The initial weights of infested and control plants were calculated on the first day of drought-stress treatment, and weight was quantified at designated time points during drought treatment. The relative plant weight declined sharply in control plants after 6 d of drought, and statistically significant differences in relative weights of infested and control plants were observed during 8 to 14 d of drought (**Fig 5B**). The relative weight of whitefly-infested plants was approximately 20% higher than that of control plants at 14 d of drought treatment (**Fig 5B**). In agreement with the higher relative weight in whitefly-infested plants after drought stress treatment, hydrogen peroxide (H_2_O_2_) was accumulated at 2 d after whitefly infestation and 8 d after drought stress in whitefly-infested plants (**Fig 5C**).

**Fig 6:**
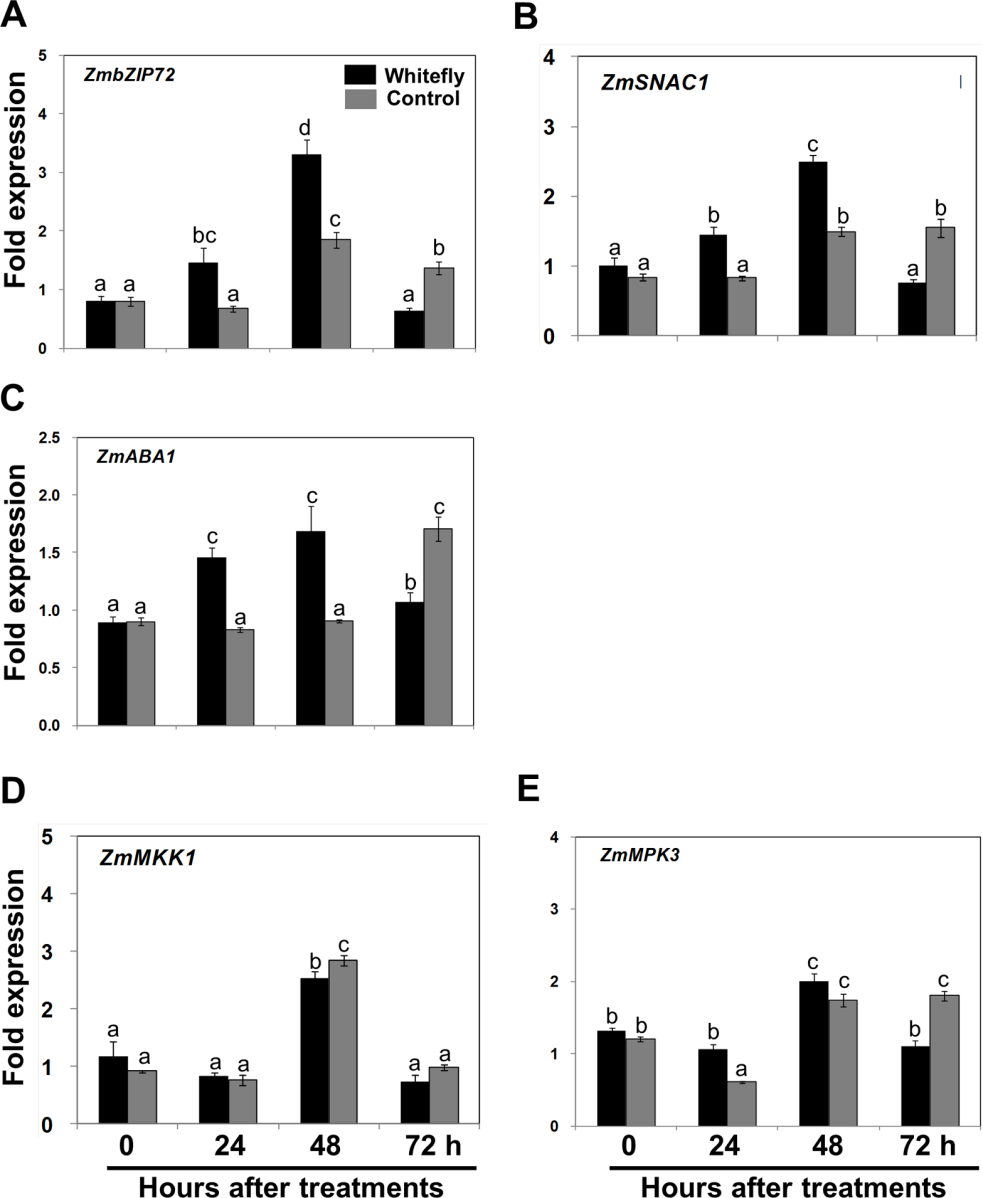
Expression patterns of drought stress-related genes in whitefly-infested roots. The V2 stage maize seedlings were infested with whitefly and then roots were harvested at designated time points (0, 24, 48, and 72 h). Expression levels of maize bZIP transcription factor *ZmbZIP72* (**A**), maize stress-responsive NAC transcription factor *ZmSNAC1* (**B**), ABA biosynthesis gene *ZmABA1* (**C**), and mitogen-activated protein kinase-related genes *ZmMKK1* (**D**) and *ZmMPK3* (**E**) were quantified and normalized with respect to *ZmGapc*. Bars represent the mean value of standard error; different letters above the graph indicate significant differences between treatments and time points (*P* = 0.05).

To assess the molecular mechanism of whitefly-induced drought tolerance, we analyzed the expression level of several genes implicated in improving drought tolerance in maize [23–27]. Two maize transcription factors, bZIP72 (*ZmbZIP72*) and stress-responsive NAC (*ZmSNAC1*), were significantly expressed in roots of whitefly-infested plants at 48 h (**Fig 6A and 6B**). The ABA biosynthesis gene *zeaxanthin epoxidase* (*ZmABA1*) transcript levels gradually increased and declined at the tested time points (**Fig 6C**). However, there were no significant up-regulation in the expression levels of two mitogen-activated protein (MAP) kinase-related genes, *ZmMKK1* and *ZmMPK3*, in whitefly-infested roots when compared to control plants (**Fig 6D and 6E**).

## Discussion

Studies on maize biotic and abiotic stress responses have focused on the elucidation of key regulators [14,15]. However, an analysis of the effects induced by piercing and sucking insects such as whitefly on maize growth, development, and drought has not been performed previously. Here, our study identified several novel results: (1) above-ground whitefly infestation stimulates below-ground root growth but not above-ground shoot growth in maize; (2) whitefly infestation enhances anthocyanin pigmentation in roots; (3) plant hormones JA/IAA and hydrogen peroxide are endogenously synthesized in maize plants; and (4) whitefly infestation copes with drought of maize. Taken together, our study provides new insights into inter-domain communications between maize and whitefly and a new understanding of herbivorous insect-mediated maize growth, development, and drought stress tolerance.

Our previous study demonstrated that whitefly infestation on pepper seedlings affects disease resistance and plant development in leaf and root [20,28]. Whitefly infestation significantly reduced plant height, although no difference in total dry weight was observed between whitefly-infested and control pepper plants [28]. By contrast, the current study did not observe differences in maize height and development between whitefly-infested and control plants (**Fig 1**). These differences may be explained by the fact that whitefly infestation differentially affects above-ground growth and development in monocots (i.e. maize) and dicots (i.e. pepper). Alternatively, the whitefly feeding density may influence plant growth and developments; approximately 50 whiteflies per leaf were feeding on pepper leaves, but only average 15 whiteflies were feeding on a maize plant. The heavier whitefly infestation may negatively influence plant shoot growth and development.

Anthocyanin derived from the phenylpropanoid pathway constitutes the red, purple, violet, and blue plant pigments [29,30] and its production has been well-studied in maize [31]. In several plant species including *Arabidopsis*, JA has a clear inductive effect on anthocyanin synthesis [32,33]. Our data also show that whitefly feeding stimulates JA and anthocyanin accumulation in maize tissues (**Figs 3 and 4C**). The total JA content in roots is approximately 25 times higher than that in leaves (**Fig 4C**). The observed stimulation of anthocyanin and JA biosynthesis in response to whitefly infestation can be explained by a recent maize study, which demonstrated that JA can positively activate anthocyanin accumulation in maize. A double mutation of two oxophytodienoate reductase genes, *ZmOPR7* and *ZmOPR8*, completely blocks anthocyanin accumulation in maize roots when compared to the corresponding wild-type, and anthocyanin pigmentation in the double mutant is restored by exogenous application of JA [14]. These results provide evidence that JA is required for anthocyanin biosynthesis in maize roots [14]. Transcript analysis shows that four out of five anthocyanin biosynthesis-related genes, *ZmCHS*, *ZmCHI*, *ZmF3H*, and *ZmUFGT*, are significantly overexpressed in whitefly-infested plants (**Fig 3B and 3C**), suggesting that these gene are likely targets for JA-mediated regulation of anthocyanin production. It is noteworthy that *ZmAOC* is clearly up-regulated in response to whitefly infestation (**Fig 4A and 4B**); this is a key upstream component of *ZmOPR7* and *ZmOPR8* in the JA biosynthetic pathway [14,15]. These results imply that anthocyanin production relies on the whitefly-mediated JA biosynthesis pathway.

There are many environmental stresses Light, UV, temperature, drought, soil salinity, air pollution, and mechanical damage are major environmental stresses that cause significant crop yield losses [34]. Abiotic environmental stresses are positively correlated with the accumulation of flavonoids such as flavonols, flavones, flavanones, flavan-3-ols, proanthocyanidins, and anthocyanins [30,35,36]. The production and crop yield of maize and soybean are seriously damaged by drought in the USA in 2012 [37], showing that drought stress is an important factor affecting maize production. Although the correlation between drought and anthocyanin accumulation in maize is not clearly elucidated after whitefly infestation in the current study, healthier plants are observed in whitefly-infested plants (**Fig 5A and 5B**). A recent study reports that plants accumulating high anthocyanin levels display the resistance to drought stress, but flavonoid-deficient anthocyanin-overexpressing plants do not [38]. These results suggest that anthocyanin may play a key role as antioxidants to scavenge reactive oxygen species (ROS) and regulate water homeostasis under drought stress. Indeed, accumulating evidence suggest that anthocyanins act as antioxidants and regulate water homeostasis [39–43].

Moreover, higher levels of hydrogen peroxide are detected in whitefly-infested plants against drought stress (**Fig 5C**). Previous reports reveal that ABA and JA are required for generating ROS and stomata closure under drought conditions; even the some key components for stomata closure can be overlapped by ABA and JA [44]. Although ABA is not detected in this study (data not shown) and the upstream signaling of hydrogen peroxide are still obscure, our results suggest that hydrogen peroxide produced by whitefly infestation plays an important role in the regulation of drought stress.

We performed expression profiling of several genes that may participate in regulating drought tolerance in maize. In particular, the maize transcription factors bZIP (*ZmbZIP72*) and stress-responsive NAC (*ZmSNAC1*) are strongly up-regulated in whitefly-infested plants (**Fig 6A and 6B**). It has been known that several maize genes belonging to bZIP and NAC transcription factors are involved in regulating stress tolerance [24,25, 45–48]. *ZmbZIP72* and *ZmSNAC1* are induced by ABA and drought treatments in seedlings, and overexpression of these genes in transgenic *Arabidopsis* confers drought tolerance and elicits the expression of ABA-inducible genes. These results suggest that these transcription factor genes are required for ABA-dependent signaling and positively modulate drought stress [24,25]. Our data show that *zeaxanthin epoxidase* (*ZmABA1*) is up-regulated in whitefly-infested plants (**Fig 6C**). In addition, two novel mitogen-activated protein kinase-associated genes (*ZmMKK1* and *ZmMPK3*) are involved in mediating drought stress in maize [23,27]. However, the transcript levels of these genes are not significantly different in whitefly-infested plants than in control plants (**Fig 6D and 6E**). Our data suggest that somehow *ZmbZIP72*, *ZmSNAC1*, and *ZmABA1* genes may participate in drought stress although the precise underlying mechanism behind is not elucidated at this moment.

**Fig 7:**
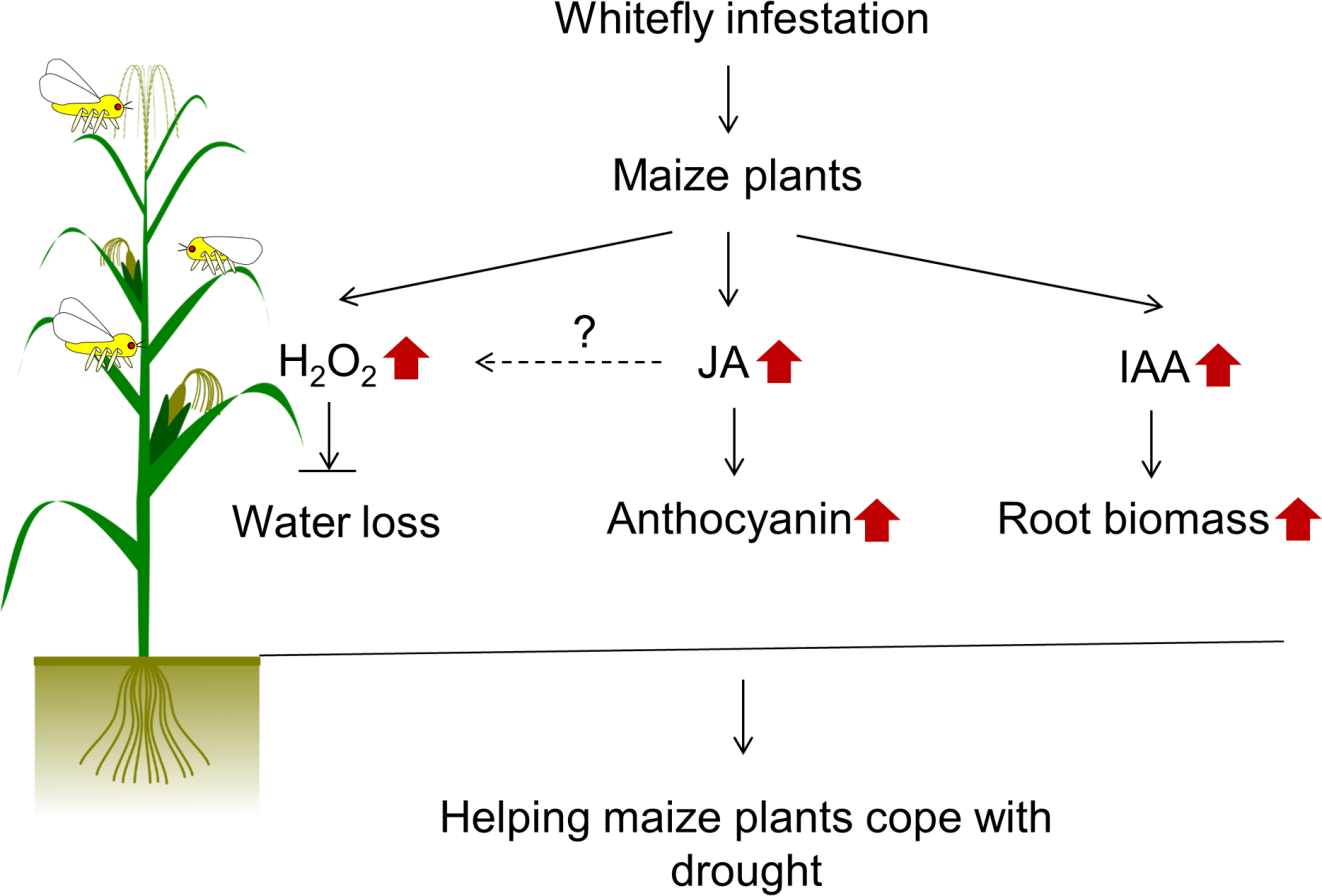
Schematic model of physiological and biochemical significance in maize plants in response to whitefly.

In conclusion, as shows in **Fig 7**, we provide new evidence that a piercing and sucking insect herbivore modulates maize growth, development, and maize-drought stress. Whitefly infestation can modulate three physiological aspects: 1) Whitefly induced JA may be implicated in activation of anthocyanin pigmentation. 2) Endogenous IAA increases total root biomass in whitefly-infested plants, 3) Accumulated hydrogen peroxide is a major factor in the regulation of drought stress. However, the underlying mechanism how JA-mediated anthocyanin accumulation affects on drought stress is still needed to further investigations. Our results clearly provide new insights into insect herbivore and drought stress. The future molecular and biochemical data may contribute to the development of field applications that can increase maize yield and production even under drought.

## Supporting Information

S1 Fig Physiological shoot phenotypes in whitefly-infested plants. The V2 stage maize seedlings were infested with whitefly for 4 weeks. Stem width was measured in whitefly-infested plants. Ten biological replicates per treatment were used for the experiment. Bars represent the mean value of standard error. Experiments were repeated three times with similar results.
(TIF)

S2 Fig Transcript profile of *ZmPAL1* and quantification of endogenous SA in leaf and root after whitefly infestation. The V2 stage maize seedlings were infested with whitefly and harvested at 0, 6, 24, 48, 72, and 96 h after infestation or control treatment. The expression levels of maize gene *ZmPAL1* was quantified by qRT-PCR in leaf (**A**) and root (**B**). The transcript level of each gene was relatively quantified and normalized using *ZmGapc*. **C**. Accumulation of endogenous SA was measured at 48 h after whitefly infestation. Bars represent the mean value of the standard error, and different letters above the graph indicate significant differences between treatments and time points (*P* = 0.05).
(TIF)

S1 Table Primer sequences used in this study. (XLSX)
