# Supplementary figures and images for: Aboveground Whitefly Infestation Modulates Transcriptional Levels of Anthocyanin Biosynthesis and Jasmonic Acid Signaling-Related Genes and Augments the Cope with Drought Stress of Maize
Source: PLoS One. 2015 Dec 2;10(12):e0143879. doi: 10.1371/journal.pone.0143879 (PMC4667997; doi:10.1371/journal.pone.0143879)

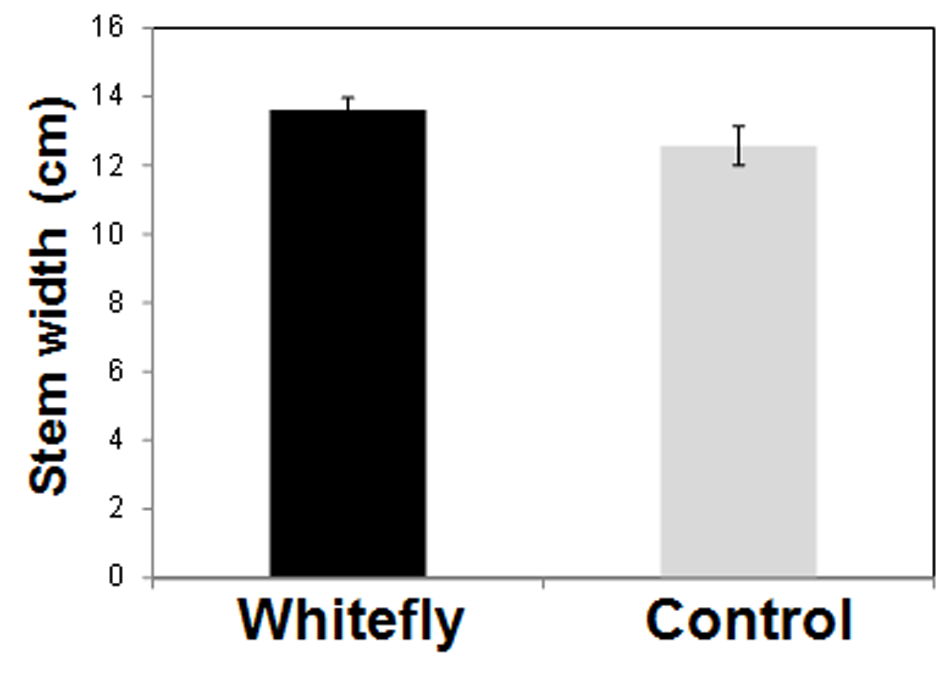

Supplement: S1 Fig — The V2 stage maize seedlings were infested with whitefly for 4 weeks. Stem width was measured in whitefly-infested plants. Ten biological replicates per treatment were used for the experiment. Bars represent the mean value of standard error. Experiments were repeated three times with similar results. (TIF) [file pone.0143879.s001.tif]

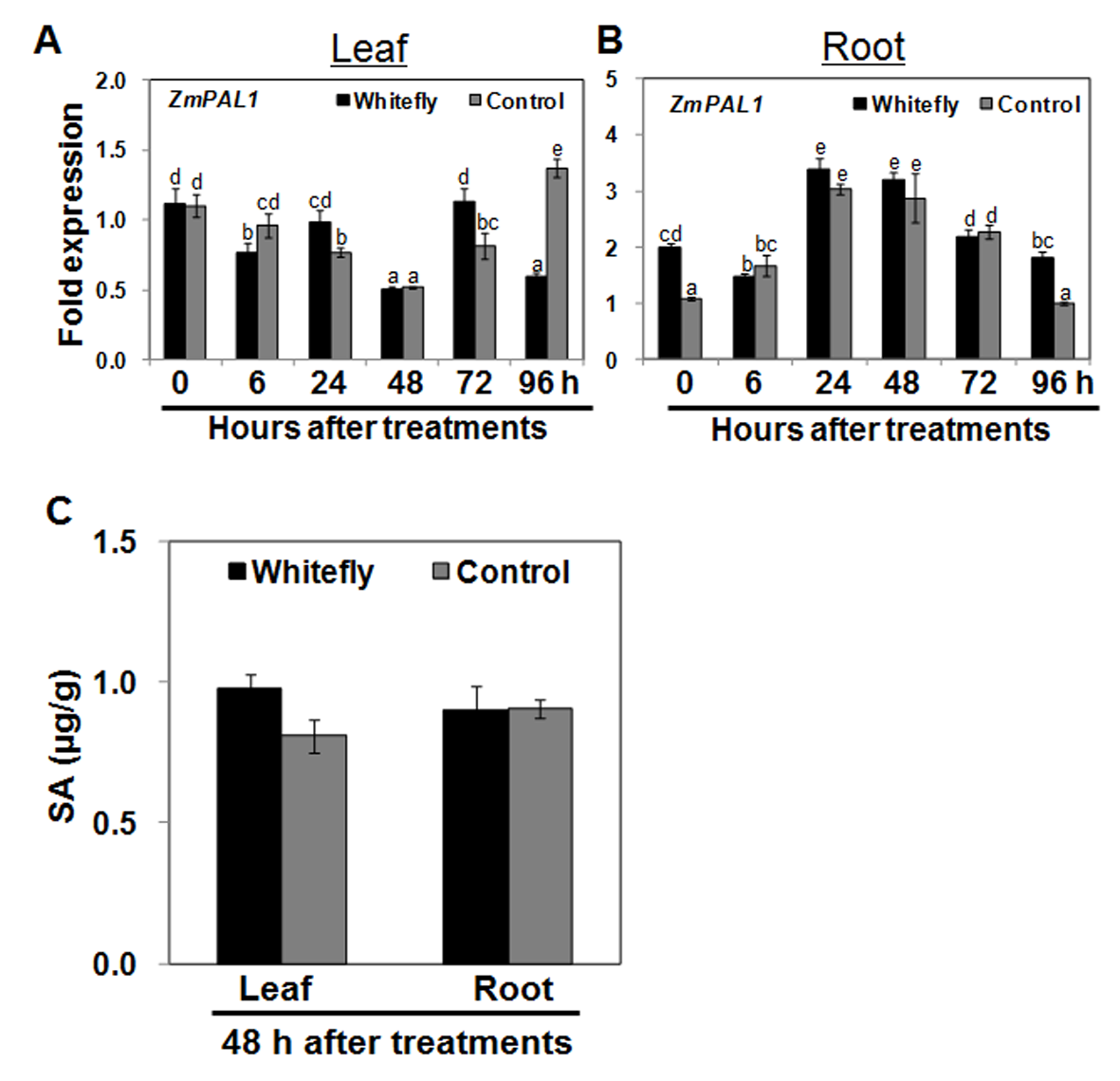

Supplement: S2 Fig — The V2 stage maize seedlings were infested with whitefly and harvested at 0, 6, 24, 48, 72, and 96 h after infestation or control treatment. The expression levels of maize gene ZmPAL1 was quantified by qRT-PCR in leaf (A) and root (B). The transcript level of each gene was relatively quantified and normalized using ZmGapc. C. Accumulation of endogenous SA was measured at 48 h after whitefly infestation. Bars represent the mean value of the standard error, and different letters above the graph indicate significant differences between treatments and time points (P = 0.05). (TIF) [file pone.0143879.s002.tif]
